# Fitting Tips and Visual Rehabilitation of Irregular Cornea with a New Design of Corneoscleral Contact Lens: Objective and Subjective Evaluation

**DOI:** 10.1155/2018/3923170

**Published:** 2018-02-01

**Authors:** Waleed Ali Abou Samra, Amani E. Badawi, Hanem Kishk, Ayman Abd El ghafar, Mohamed M. Elwan, Hossam Youssef Abouelkheir

**Affiliations:** Mansoura Ophthalmology Center, Mansoura University, Mansoura, Egypt

## Abstract

**Objectives:**

To study the fitting and the visual rehabilitation obtained with a corneoscleral contact lens, namely, Rose K2 XL in patients with irregular cornea.

**Methods:**

This prospective study included 36 eyes of 36 patients with irregular cornea fitted with Rose K2 XL. Refractive and visual outcomes and mesopic and aberrometric parameters of fitted eyes were assessed at 2 weeks, 3 months, and 6 months after the initial lens use. Objective and subjective parameters of patient satisfaction and lens comfort were noted. Causes of lens discontinuation and complications were also recorded.

**Results:**

Average logMAR VA improved significantly from 0.95 ± 0.09 without correction to 0.04 ± 0.05 six months after lens wear. Similarly, mesopic and aberrometric measures were significantly improved. Statistical analysis of the subjective patients' responses showed a significant acceptance of the lens by most of them. At the end of follow-up, the mean wearing time was 9.9 ± 2.9 hours per day. The most common cause of wearing discontinuation was persistent discomfort (16.7%) and high lens expenses(16.7%). Self-assessed questionnaire showed statistically significant improvement in nearly all measured subjective parameters.

**Conclusion:**

Rose K2 XL lenses provide patients with irregular cornea with both quantitative and qualitative optimal visual function with high degree of patient comfort and satisfaction.

## 1. Introduction

Since irregular astigmatism produces pronounced visual deterioration, the refractive surgeons, for decades, have sought an archetype solution to manage such error. Several surgical interventions can be considered to improve the corneal irregularities as thermocauterisation, intrastromal corneal rings, and many varieties of corneal transplant [1–4]. So far, none of these procedures reliably accomplish an ideal visual outcome. Therefore, surgical intervention should be the last option as far as possible.

Various kinds of contact lenses are deemed one of the nonsurgical approaches to the correction of irregular high astigmatism that cannot be corrected with spectacles [5–8]. Rigid gas permeable (RGP) lens might be the best choice as it has high oxygen permeability, provides good visual acuity (VA), and carries a lower incidence of corneal neovascularization and infective keratitis comparing with the soft contact lenses [9–12].

However, excessive lens movement in those patients might lead to visual fluctuation, ocular discomfort, and impaired corneal physiology. Therefore, choosing the appropriate design and method of lens fitting in a way that maintains the patient with an acceptable tolerance together with adequate vision is challenging.

Scleral contact lens (ScCL) seems to be more effective in irregular corneas than traditional corneal RGP lens since it provides an effective option for correction of residual ametropia and high-order aberrations (HOAs) and masking surface corneal irregularities with the tear lens between the posterior lens surface and anterior corneal surface [13–15].

Most of the previous studies measured the clinical performance of the ScCL in terms of improvement in VA [13, 15–17]. To the best of our knowledge, this is the first study reporting the objective and subjective results of a new design of corneoscleral contact lens namely Rose K2 XL in cases of irregular corneas with a recording of its role in correcting of mesopic vision and HOAs. Moreover, fitting tips, patient satisfaction, and complications of such lenses were demonstrated.

## 2. Subjects and Method

This prospective study was carried out on randomly chosen 36 eyes of 36 patients with irregular astigmatism of different etiologies. The patients were enrolled from outpatient clinics of Mansoura Ophthalmic Center, Mansoura University between 2015 and 2016. The inclusion criterion accepted all irregular astigmatisms unable to achieve a good VA with spectacles or standard design of soft or corneal RGP contact lenses.

Patients with severe eye dryness, active ocular disorders, and illiterate ones were excluded from the study. The study was reviewed and approved by the institutional research board (IRB) of the Mansoura Faculty of Medicine, and was adhered to the tenets of the Declaration of Helsinki. The study protocol, the fitting of the lenses, and study consequences were clarified to the patients. All participants have signed informed consents to be enrolled in the study.

The causes of irregular astigmatism were keratoconus, pellucid marginal degeneration (PMD), post-LASIK ectasia, and irregular corneas following keratoplasty, intrastromal rings, and corneal traumatism.

Before fitting the corneoscleral lenses, we performed a complete ophthalmological examination, including measurement of uncorrected and best-corrected spectacle visual acuity (UCVA and BCSVA) using logMAR charts, anterior and posterior segments examination, applanation tonometry, and corneal topography with Scheimpflug topographic system (Pentacam, OCULUS Optikgeräte GmbH, Wetzlar, Germany).

Mesopic vision tests and ocular aberrations were assessed using Mesotest II (OCULUS Optikgeräte GmbH, Wetzlar, Germany) and Ocular aberrometry (Zywave II, Bausch and Lomb, Munich, Germany), respectively. Subjective vision-related questionnaire was also achieved.

### 2.1. Fitting of the Lens and Follow-Up Schedule

After the confirmation of eligibility criteria at the first visit, eyes were fitted with aspheric corneoscleral contact lens, namely, Rose K2 XL after measuring precisely the lens parameters.

Rose K2 XL lenses are made of tisifilcon A (Menicon Z; Menicon Co. Ltd, Nagoya, Japan) with high permeability Dk value (Dk = 163). They have an aspheric back optical surface at which optical zone decreases as the base curve steepens. This corneoscleral lens design with its large diameter (13.6–15.6 mm) and corneal vaulting is used for keratoconus, PMD, keratoglobus, LASIK-induced ectasia, postkeratoplasty, and any irregular corneal condition that cannot be successfully treated by fitting a lens within the limbus.

Contact lens fittings were performed according to the manufacturers' guidelines by the same experienced doctor. The diagnostic fitting set is an incorporated set composed of sixteen lenses with base curves (BC) ranged from 6.00 to 8.00 mm and the overall diameter of any lens in the set is 14.6 mm with a standard edge lift designing. The lens power gradually changing to approach the final lens power. However, we can request the lens with a diameter ranged from 13.6 mm up to 15.6 mm, BC ranged from 5.80 to 8.40 mm, with any power and with various traits of edge lift.

Adherence to the guidelines of CL fitting, the first lens was chosen for each eye in accordance with the eye parameters and pathologies; in postgraft and post-LASIK, 0.7 mm steeper than mean K's; in PMD, 0.6 mm steeper than mean K's; in corneal rings, 0.1 mm steeper than mean K's; and in keratoconus, 0.2 mm flatter than mean K's.

A preservative-free sterile saline solution was instilled with sodium fluorescein (Haag-Streit AG, Koeniz, Switzerland) into the concave side of the lens. The central fit was immediately judged after insertion and a progressive flatter or steeper BC was selected accordingly till a light feathery touch was achieved at the highest point on the cornea. The lens was allowed to settle for a further 20 minutes and the fit was reevaluated. If further fluorescein is required, it was placed on the sclera at 12, just above the lens and the patient was asked to blink several times. If fluorescein did not circulate behind the lens, we manipulated the lower and/or the upper edge to encourage fluorescein to flush under the lens.

Once the correct central fit has been achieved, the edge lift was evaluated 0.8 to 1 mm wide. If the band is too wide, it may show liftoff and bubbling at the edge of the lens with associated discomfort, so decreasing the edge lift was recommended. On the other hand, if the band is too narrow or edge lift is tight with blanching of the conjunctival vessels from the limbus to the edge of the lens and/or hyperemia to conjunctival vessels just outside the lens, we should increase the lift.

As regards the diameter, the lens should extend 1.3 to 1.5 mm outside the limbus and sit evenly around it. However, a decentered apex might cause the lens to locate inferiorly so increased diameter and/or steep BC could improve centration.

On the first insertion, the lens should move slightly on blinking (maximum of 0.5 mm). The movement was judged at 6 o'clock by asking the patient to look up and blink. The excessive movement might cause patients discomfort, so decreasing the edge lift, steepening the BC, increasing the diameter, or a combination of these could help to decrease the lens movement. Opposite parameter changes were applied to increase the movement. Once the appropriate lens parameters were chosen, an accurate overrefraction was performed and the final lens was ordered from the manufacturer.

After the arrival of the ordered lenses, patients wore them and were evaluated on slit lamp in outpatient clinics. If the lenses gave acceptable fit, vision, and comfort, all instructions were given to the patients including the proper way of lens insertion and removal, cleaning, and wearing schedule. Moreover, follow-up regimen was given to the patients. If the ordered lenses were not acceptable, the lenses were reordered with the appropriate changes and the patient was scheduled for another dispensing visit when the new lenses arrived.

### 2.2. Patient Data

The collected descriptive and clinical data were age, gender, laterality of the disease, UCVA, BSCVA, subjective refraction (sphere and cylinder), keratometric measures (Kmax and Kmin), and corneal astigmatism. The collected data of lens fitting were the power of lens, diameter, base curve, edge lift, number of trial for the optimal fit, and the frequency of refitting during the follow-up period. For an optical performance and visual outcome estimation, over-refraction, best contact-corrected visual acuity (BCCVA), contrast sensitivity, and glare levels were measured at 2 weeks, 3 months, and 6 months following wearing of contact lenses. Additionally, total root mean square (RMS) and higher order aberration (HOA) were recorded and appraised for a 6 mm pupil using the high-resolution aberrometer.

We assessed patient comfort and satisfaction with the lenses during the follow-up visits according to simple parameters used in one of our previously published study [5]. Using a 10-point scale, we recorded the self-reported assessment of dryness and comfort giving 0 corresponding to severe dryness/excessively uncomfortable and 10 corresponding to no dry sensation/no awareness. Furthermore, maximum daily comfortable wearing time (CWT), the patient predilection for keeping to wear his contact lens, number of lens removals a day, and causes of discontinuing of lens wearing were recorded. Postfitting complications as allergic conjunctivitis, ocular dryness, superficial punctate keratitis (SPK), and scratch or break of the lens were also recorded.

### 2.3. Subjective Analysis

In the current study, the subjective evaluation was fulfilled by requesting the participants to fill in a previously prepared questionnaire just before the CL fitting and after 6 months. It covered the following: visual clarity, visual annoyance symptoms (such as glare, diplopia, starburst, halos, and visual fluctuation), near and far glasses dependence, restrictions of daily and social activities (such as shopping, cooking, driving, TV watching, and others), and patient satisfaction. The collected data from the questionnaire was then scored. The annoying visual symptoms were scored on a scale from 1 (none) to 5 (severe), patient satisfaction and clarity of vision were also ranked from 1 (none) to 5 (excellent), whereas glasses dependence was scored by asking how often patients used glasses for distance and near vision; (never 1, rarely 2, on occasion 3, often 4, and always 5). The data of subjective outcomes were displayed as the mean score for each of the subjective queried parameters.

### 2.4. Contrast and Glare Sensitivity Test

It was implemented with a Mesotest II (OCULUS Optikgeräte GmbH, Wetzlar, Germany), which consists of Landolt rings of various grades of contrast located in front of a low-luminosity backdrop. There are four different levels of contrast: 1 : 23/1 : 5/1 : 2.7/1 : 2 which constitute the ratio between the luminosity intensity of the optotypes and the backdrop. There are 8 tests (4 without and 4 with glare). Test 1, with contrast level 1 : 23, is the most easily recognized. For statistical analysis, each level of the glare or the contrast test was given a score ranging from 20% to 100% at the 1 : 23 level and at the 1 : 2 level, respectively.

### 2.5. Statistical Analysis

The recorded data were analyzed by statistical package SPSS version 15.0 for Windows (SPSS Inc., Chicago, IL). The nonparametric Wilcoxon Rank Sum test was applied to assess the significance of differences between pre and postfitting data in each visit. The level of significance used was always the same (*p* < 0.05). VA measurements were calculated as logMAR.

## 3. Results

The study was carried out on 36 eyes of 36 patients (20 male and 16 female) with an average age 32.4 ± 10.8 years. The study included eyes with high degree of irregular astigmatism due to keratoconus in 10 eyes (27.8%), postkeratoplasty in 7 eyes (19.4%), PMD in 6 eyes (16.7%), post-LASIK ectasia in 5 eyes (13.8%), postcorneal rings in 4 eyes (11.1%), and corneal injuries in 4 eyes (11.1%). Demographic information of studied patients is presented in Table 1.

Tables 2 and 3 display the whole fitting parameters and characteristics of Rose K2 XL lenses. The average base curve of the used lenses was 7.7 ± 0.22 mm (range, 7.4 to 8.00 mm). The average power was −3.80 ± 2.0 D (range, 0.00 to −6.50). Only lenses with a standard diameter (14.60 mm) and edge lifts were used in this study. An average of 1.8 ± 1.2 trial lenses were used (range, 1 to 3 lenses) till final fitting. A lens refitting was required in only one patient during the follow-up period because of a broken lens.

VA measures were reported as logMAR values. There was a highly significant improvement in VA after fitting with Rose K2 XL lenses. BCCVA was 0.05 ± 0.03 two weeks after fitting in comparison to UCVA (0.95 ± 0.09) and BSCVA (0.65 ± 0.17) (*p* < 0.001). There was a significant improvement in both astigmatic and spherical errors. It improved significantly from −4.3 ± 1.8 D and −4.8 ± 1.2 D before fitting to −0.31 ± 0.27 D and −0.38 ± 1.1 D after fitting (*p* < 0.001). Improvement of all values remained significant during follow-up periods (Table 4).

As regards the quality of vision, usage of the contact lenses led to a significant improvement in glare and contrast sensitivity tests. They increased from prefitting uncorrected values of 41.6% ±7.2 and 39.6% ±6.9, respectively, to postfitting corrected values of 81.9% ±5.9 and 69.9% ±6.2 (*p* < 0.001). Similarly, aberrometric measures demonstrated significant reduction of both total RMS and HOA RMS values after contact lenses fitting (*p* < 0.001). Improvement of all measures was recorded in each follow-up visit (Table 5).

Most of the patients exhibited strong satisfaction and robust acceptance of the lens usage. The maximum comfort wearing time (CWT) a day increased progressively through the follow-up visits to nearly 9.9 ± 2.9 hours at the last visit. Using a 10-point scale, the self-assessment of comfort and dry eye sensation revealed a significant degree of comfort for the corneoscleral lenses designing (8.2 ± 1.4 and 7.9 ± 1.1, respectively, after 6 months). The average number of lens removal per day was 2 ± 1. By the last visit, 30 patients (83.3%) reported a preference to continue using their contact lenses, while only 6 patients (16.7%) asked for an alternative to their contact lenses (Table 6).

Table 7 shows the causes of the lens discontinuation. The most common cause was “persistent discomfort,” followed by “high lens expenses,” and lastly “handling difficulties.”

Fitting problems in eyes are recorded in Table 8. These included, in order of frequency, allergies (11.11%), tight lens syndrome (11.11%), dry eye syndrome (8.33%), broken lenses (5.56%), lens scratches (2.78%), and superficial punctate keratitis (2.78%). Corneal edema, neovascularization, corneal ulcers, or pannus were not recorded throughout the study.

Patient self-assessed subjective parameters and measures of patient satisfaction with Rose K2 XL were displayed in Table 9. There was a statistically significant improvement in nearly all recorded parameters.

## 4. Discussion

Correcting corneas with irregular astigmatism is a challenge for the ophthalmologist. In most cases, spectacles with adequate correction failed to improve the VA, leaving contact lenses as the sole option for treatment before the surgery [7, 18, 19]. Such group of patients generally has severe disease in which standard soft or RGP lenses are not viable options for good vision or patient comfort. Fitting of such challenging corneas with small overall diameter spherical RGP lenses is associated with exaggerated lower lens edge standoff, unsteady centration, ghosting, and consequent narrower fields of view. Also, it could lead to lens dislocation and even lens loss when blinking [20–22].

Corneoscleral lenses with an improved peripheral fit and larger diameter allow for a better alignment of the peripheral cornea and reduced edge standoff, which discourages lens dislocation and provides the patients with adequate comfort and optimal vision [13–15, 23]. As demonstrated by our experience, ScCL can delay and even prevent the need for surgery, which carries risks of infection, rejection, and postoperative severe astigmatism [24–27].

To our knowledge, this is the first published report of prospective outcomes describing fitting tips, quantitative and qualitative visual measures, safety, and subjective outcomes with Rose K2 XL lens in different causes of irregular corneas.

In the current study, our results demonstrated marked improvement in VA in all studied patients. BCCVA was 0.05 ± 0.03 after fitting in comparison to UCVA (0.95 ± 0.09) and BSCVA (0.65 ± 0.17) (*p* < 0.001). Considering the same design of contact lenses used in our study (Rose K2 XL), Romero-Jiménez and Flores-Rodríguez [28] observed an improvement in VA from 0.14 logMAR with the patient's usual contact lens to 0.10 logMAR with the Rose K2 XL scleral lens (*p* = 0.079). Another study [15] also noted statistically significant improvement, although patients had a poorer VA with their usual lenses than in the study of Romero et al. An earlier study conducted by Stason et al. [29] demonstrated that VA did not improve in all patients fitted with Boston ocular surface prosthesis (PROSE lenses), although none obtained poorer results. In our study, however, VA improved in all eyes after fitting with the Rose K2 XL lenses. This difference may be attributable to the type of lens used or to the sample size, which was not very large in our study. Similar results were demonstrated in keratoconic patients wearing Rose K lens design [30–32].

Currently, there was an additional significant improvement in the visual quality beside to the remarkable improvement in VA. To the best of our knowledge, this is the first paper reporting the mesopic and aberrometric measures after fitting with Rose K2 XL lenses. Both glare and contrast sensitivity tests exhibited significant improvement with the usage of the lenses. They increased from prefitting uncorrected values of 41.6% ±7.2 and 39.6% ±6.9, respectively, to postfitting corrected values of 81.9% ±5.9 and 69.9% ±6.2 (*p* < 0.001). Similarly, aberrometric measures demonstrated a significant reduction of both total RMS and HOA RMS values after contact lenses fitting (reduction of 72.5% in HOA) (*p* < 0.001). A study carried by Porcar et al. [33] also demonstrated an important reduction of total HOAs (an average of 78% reduction) after wearing of multiaspheric geometry design in eyes with irregular corneas after laser-assisted in situ keratomileuses (LASIK) surgery. In previous studies, Gemoules and Morris [34] found a reduction in the total HOAs of 66% in eyes fitted a reverse geometry corneoscleral lens design. Tan et al. [35] fitted a spherical RGP on a pupil size of 6 mm and found a reduction in the total HOAs of 69%.

The unique design of Rose K2 XL lens with aspheric back optical zone, front surface aberration control, and precise edge lift options contributes to the centration and lens stabilization in the eye and reduces the HOAs and blur outcoming with the use of standard soft or RGP lenses. Additionally, with the suggested apical clearance fit of the lens, the tear layer can correct corneal irregularity decreasing aberrations and providing crisp, clear, and consistent vision. After 6 months wearing of Rose K2 XL lenses, no statistically significant differences were found in VA, mesopic vision, or total HOAs in regard to the initial fitting. This could be due to the stability of contact lenses parameters and maintenance of an appropriate corneal tear film.

Most of the patients showed a robust admittance of the lens. The maximum wearing time per day was 9.9 ± 2.9 hours at the end of follow-up period. Self-reported estimate of comfort and dryness confirmed a high degree of comfort using the lenses (8.2 ± 1.4 and 7.9 ± 1.1, resp.). Thirty patients (83.3%) expressed a preference to continue wearing their contact lenses at the last follow-up visit, while only 16.7% of patients asked for another option.

Our results are consistent with a prior report that revealed a significant degree of comfort in patients wearing this design of lenses for a mean of 9 hours a day [15]. Also, the results go hand in hand with previously reported data about Rose K lenses that indicates a high degree of comfort in keratoconus patients wearing their lenses for more than 10 hours daily [30–32]. However, wearing time could be increased in some patients up to 15 hours per day by instructing them to remove the contact lens every few hours and refill them with fresh preservative-free sterile saline solution as it happened in Ortenberg et al. [36].

However, 6 patients (16.7%) in the present study recorded ocular discomfort and this was the main cause for discontinuing use of their lenses during the follow-up period. All studied patients were examined regularly, so that patient instructions with a removal of lenses for few hours during the waking period and use of tear substitute drops and/or lubricant gel exceedingly decreased the symptoms in 4 patients. While two patients did not respond to any treatment and thus ceased wearing their lenses by the end of follow-up period. The cost price of the lenses was one of the prime patients' complaints as the lenses are not covered under government health insurance in Egypt and the expense of contact lenses is roughly twice the price of standard RGP lenses (like Rose K lens).

Earlier studies of corneoscleral lenses stated that the difficulty of lens handling was one of the main problems comparing to standard corneal RGP lenses and thus was a chief reason for lens discontinuation [15, 37]. Four patients (11.1%) in our study reported problems with handling. These patients were given instruction at each visit about the proper way of insertion and removal of their lenses and motivated to use the special suction holder for this issue.

Table 8 shows the complications recorded in studied eyes. In our study, allergies occurred in 11.1% of the patients compared to higher incidence occurred with other designs of contact lenses [38]. Patients reported irritating symptoms such as ocular discomfort, foreign body sensation, and itching that occasionally increased after lens discontinuation. Such annoying complication is believed to occur due to the prolonged wearing times, hypersensitivity to CL deposits or its care solutions, and lens-induced mechanical microtrauma. It is typically distinguished by small papillae and hyperemia of the superior tarsal conjunctiva. Topical mast cell stabilizers/antihistamine drops (before and after CL usage) is the proper medication as well as lubricant agents and mild topical steroids could be useful in some cases.

In this study, we fitted all eyes with standard edge lift lenses as we did not guarantee the patient comfort with different edge lifts, especially we did not have trial lenses with increased or decreased edge lifts. Moreover, a tight edge on initial insertion gives much better comfort than a loose edge but may cause issues in the long term. This is why we have a relatively high percentage of the tight lens syndrome in our study (11.11%). However, refitting with the increased edge lift design was recommended to solve the patient's problem. Dry eye occurred in 3 patients who instructed to use preservative-free lubricants. 2 lenses were broken because of mechanical trauma while one lens had scratches from bad patient manipulation of their lenses.

The new materials with high oxygen permeability and reduced central thicknesses of ScCL may resolve or diminish the incidence of corneal edema. Also, tear interchange that is obtained by a fitting technique with smaller diameter lenses and apical clearance might be accountable for preserving an adequate corneal physiology. These favorable factors that are present in Rose K2 XL show that these contact lenses are proposed as a safe and effective procedure fitted on subjects with irregular corneas as those having ectasia following LASIK surgery [33]. Our results confirmed this hypothesis; as no any case of pannus, neovascularization, or corneal edema was recorded in the current study, at the very least through the proportionally short-term follow-up.

Precise edge lift control of such design with the lens movement and how easily fluorescein enters the lens at 6 o'clock with upward pressure ensure corneal safety, so no corneal staining was demonstrated in our fitted eyes. As regards the infection, no case of infectious keratitis was observed which agreed with previously reported results [13, 15, 33, 37]. However, only one eye suffered from SPK but this was not considered to be a vision-threatening consequence.

In a previous study, four of 14 eyes developed bacterial keratitis, however in this study, the ScCL were worn continuously, and the fluid compartment contained both antibiotic and steroid, which might raise the vulnerability of microbial keratitis [39].

As regards patient self-assessed parameters, all measured subjective parameters revealed statistically significant amelioration, with sufficient patient satisfaction. This is ascribed to the clarity of vision, glare decrease, spectacles independence, and ability to implement daily activities. Using a questionnaire, Segal et al. [39] reported marked betterment in the life quality in more than 80% of patients. Likewise, Romero-Rangel et al. [40] recorded that about 92% of participants showed marked improvement in daily performance of their activities reflecting the improvement in their life quality. Such results go hand with hand with that recorded in patients wearing Rose K lenses [30–32].

As a conclusion of our study, innovative design of corneoscleral contact lenses, namely, Rose K2 XLcan be a useful tool in fitting of challenging corneas with irregular astigmatisms such as KC, PMD, LASIK-induced ectasia, post-PRK, and posttraumatic cases in which the medical treatment and/or traditional CLs could not achieve the desired visual outcome, and the surgical correction is contraindicated or undesirable. They give a remarkable improvement in both quantity and quality of visual acuity, with a high degree of safety, comfort, stability, and fewer complications. However, larger sample size, longer follow-up, and evaluation with a validated questionnaire are recommended in future studies.

## Figures and Tables

**Table 1:** Demographic data of the participants in the study.

Parameter	Values
Number of patients (eyes)	36 (36)
Mean age ± SD, years	32.4 ± 10.8
Gender (male/female)	20/16
Keratometry, D	
K max	51.5 ± 3.7
K min	44.1 ± 2.6
Mean corneal astigmatism ± SD, D	−5.1 ± 1.3
Mean refractive cylinder ± SD, D	−4.8 ± 1.2
Mean refractive sphere ± SD, D	−4.3 ± 1.8
MRSE	−6.9 ± 1.7
Causes of irregular cornea, number (%)	
Keratoconus	10 (27.8%)
Postkeratoplasty	7 (19.4%)
PMD	6 (16.7%)
Post-LASIK ectasia	5 (13.9%)
Post-intrastromal rings	4 (11.1%)
Posttrauma	4 (11.1%)

D: diopter; MRSE: mean refractive spherical equivalent.

**Table 2:** A summary of the main characteristics of the Rose K2 XL contact lenses.

Material	Menicon Z
	Boston XO
Diameter	13.6–15.6 mm
	Standard diameter = 14.60 mm.
Base curves	5.80–8.40 mm
Sphere powers	Varies depending on material
Edge lift	5 standard lifts (optimal fit in 90% of cases)
	Total of nine edge lift options are available

**Table 3:** Rose K2 XL lens parameters in studied patients.

Overall diameter (mm)	14.60
Base curve (mm)	7.7 ± 0.22 (7.4 to 8.00)
Power (diopter)	−3.80 ± 2.0 (00 to −6.50)
Edge lift	Standard
Ease of fitting (number of trial lenses)	1.8 ± 1.2 (1 to 3)
Number of lenses refit	1

**Table 4:** Visual and refractive measures (mean ± SD) before and after fitting with Rose K2 XL lenses during follow-up periods.

Parameter	Prefitting	Postfitting					
		2 weeks	*p* value^a^	3 months	*p* value^b^	6 months	*p* value^c^
UCVA	0.95 ± 0.09	0.95 ± 0.08	0.35	0.93 ± 0.11	0.29	0.92 ± 0.10	0.19
BSCVA	0.65 ± 0.17	0.64 ± 0.18	0.22	0.63 ± 0.17	0.19	0.62 ± 0.19	0.13
BCCVA		0.05 ± 0.03	**<0.001** ^∗^	0.04 ± 0.05	**<0.001** ^∗^	0.04 ± 0.05	**<0.001** ^∗^
Sphere (D)	−4.3 ± 1.8^(1)^	−0.31 ± 0.27^**(2)**^	**<0.001**	−0.33 ± 0.31^**(2)**^	**<0.001**	−0.32 ± 0.44^**(2)**^	**<0.001**
Cylinder (D)	−4.8 ± 1.2^**(1)**^	−0.38 ± 1.1^**(2)**^	**<0.001**	−0.36 ± 1.3^**(2)**^	**<0.001**	−0.36 ± 1.2^**(2)**^	**<0.001**
MRSE (D)	−6.9 ± 1.7^**(1)**^	−0.51 ± 0.22^**(2)**^	**<0.001**	−0.53 ± 0.27^**(2)**^	**<0.001**	−0.54 ± 0.25^**(2)**^	**<0.001**

UCVA: uncorrected visual acuity; BSCVA: best spectacle corrected visual acuity; BCCVA: best contact-corrected visual acuity (logMAR measures); D: diopter; MRSE: mean refractive spherical equivalent. Postfitting *p* values (a, b, and c) derived from posthoc analysis. ^a^Comparing 2 weeks after CL with prefitting values. ^b^Comparing 3 months after CL with prefitting values. ^c^Comparing 6 months after CL with prefitting values. ^1^Without contact lenses or glasses. ^2^With contact lens. ^∗^Comparison between the BSCVA and the BCCVA. Significant at *p* ≤ 0.05.

**Table 5:** Aberrometric parameters and mesopic vision testing results (mean ± SD) before and after fitting with Rose K2 XL lenses during follow-up periods.

Parameter	Prefitting	Postfitting					
		2 weeks	*p* value^a^	3 months	*p* value^b^	6 months	*p* value^c^
Contrast Sensitivity (%)	41.6% ±7.2^**(1)**^	81.9% ±5.9^**(2)**^	<0.001	83.7% ±5.2^**(2)**^	<0.001	83.9% ±6.1^**(2)**^	<0.001
Glare (%)	39.6% ±6.9^**(1)**^	69.9% ±6.2^**(2)**^	<0.001	71.1% ±6.4^**(2)**^	<0.001	71.9% ±6.9^**(2)**^	<0.001
Total RMS	4.9 ± 1.55^**(1)**^	2.2 ± 0.62^**(2)**^	<0.001	2.1 ± 0.59^**(2)**^	<0.001	2.00 ± 0.60^**(2)**^	<0.001
HOA RMS	1.2 ± 0.57^**(1)**^	0.34 ± 0.27^**(2)**^	<0.001	0.34 ± 0.23^**(2)**^	<0.001	0.33 ± 0.21^**(2)**^	<0.001

Total RMS: total root mean square; HOA RMS: high order aberration root mean square. Postfitting *p* values (a, b, and c) derived from posthoc analysis. ^a^Comparing 2 weeks after CL with prefitting values. ^b^Comparing 3 months after CL with prefitting values. ^c^Comparing 6 months after CL with prefitting values. ^1^Without contact lenses or glasses. ^2^With contact lens. Significant at *p* ≤ 0.05.

**Table 6:** Patient satisfaction in the wearing group.

Parameter	2 weeks	3 months	6 months
CWT (hours)	9.3 ± 2.4	9.7 ± 3.3	9.9 ± 2.9
Comfort score	7.8 ± 1.5	8.0 ± 1.7	8.2 ± 1.4
Dry score	7.7 ± 1.3	7.7 ± 1.2	7.9 ± 1.1
Number of lens removal per day	2 ± 1	3 ± 1	2 ± 1
Patient preference	80.6%	83.3%	83.3%

CWT: comfort wearing time.

**Table 7:** Reasons for discontinuing lens wearing.

Cause	Number of patients (%)
Discomfort	**6 (16.67%)**
Cost	**6 (16.67%)**
Handling difficulties	**4 (11.11%)**

**Table 8:** Complications in eyes fitted with the Rose K2 XL lenses.

Complication	Number of eyes (%)
Allergies	**4 (11.11%)**
Tight lens syndrome	**4 (11.11%)**
Dry eye	**3 (8.33%)**
Broken lens	**2 (5.56%)**
Lens scratches	**1 (2.78%)**
Superficial punctate keratitis	**1 (2.78%)**

**Table 9:** Patient self-assessed mean subjective ratings before and 6 months after fitting with Rose K2 XL (mean ± SD).

Parameter	Prefitting	Postfitting	*p* value
Clarity of vision	2.17 ± 1.1	4.2 ± 0.92	0.003
Patient satisfaction	2.1 ± 1.3	4.47 ± 0.75	0.002
Visual fluctuation	3.23 ± 0.98	1.1 ± 0.33	0.01
Glare	2.66 ± 0.81	1.13 ± 0.75	0.008
Halo	3.67 ± 1.41	1.51 ± 0.66	0.005
Starburst	3.22 ± 0.57	0.94 ± 0.59	0.002
Diplopia	0.98 ± 0.59	0.73 ± 0.47	0.06
Activity limitations^∗^	4.13 ± 1.53	1.28 ± 0.78	<0.001
Far spectacle dependence	4.67 ± 0.99	0.83 ± 0.55	<0.001
Near spectacle dependence	3.33 ± 1.77	2.68 ± 1.43	0.05

^∗^Activities: includes reading, driving, playing games, cooking, and watching the TV.
